# miR-335-3p acts as a tumor suppressor in esophageal squamous cell carcinoma and predicts favorable prognosis

**DOI:** 10.1186/s41065-026-00702-w

**Published:** 2026-07-21

**Authors:** Miao He, Shuojun Lu, Feng Yang, Jiangqi Xu, Ran Zhu

**Affiliations:** 1 Gastrointestinal Oncology Unit, Clinical Center for Digestive Diseases, People’s Hospital affiliated to Chongqing Three Gorges Medical College, Chongqing, 404000 China; 2 The First Department of Oncology, Xinxiang Central Hospital, Fourth Clinical College of Xinxiang Medical University, Xinxiang, 453000 China; 3 Department of Geriatrics, Jiujiang First People’s Hospital, Jiujiang, 332000 China; 4 Department of Pathology, Dongguan Songshan Lake Central Hospital, No.1 Xianglong Road, Shilong Town, Dongguan, 523000 China

**Keywords:** ESCC, miR-335-3p, GFPT1, Prognosis, Cell biological function

## Abstract

**Background:**

Esophageal cancer is a highly invasive malignancy that severely impairs normal digestive function and poses a substantial threat to patient survival. The pathogenesis of miRNA-mediated tumors has been widely documented.

**Aim:**

Verifying the involvement of miR-335-3p in the pathogenesis of esophageal squamous cell carcinoma (ESCC).

**Methods:**

The study enrolled 90 ESCC patients, from whom clinical data and pathological tissue samples were acquired. The prognostic potential of dysregulated miR-335-3p in ESCC was assessed using the Kaplan-Meier method. miR-335-3p and GFPT1 expression in the specimens were measured by RT-qPCR. Cellular biological functions were verified through transfection, CCK-8, Transwell, and kit-based assays. The targeting relationship was ascertained by luciferase activity assays.

**Results:**

miR-335-3p was downregulated in ESCC, which is indicative of poorer prognostic outcomes. GFPT1 was up-regulated and was regarded as a target of miR-335-3p. Increased miR-335-3p levels markedly impaired cellular biological functions. Conversely, simultaneous overexpression of GFPT1 alleviated the negative effects induced by miR-335-3p mimic, which was associated with the partial restoration of cell activity and antioxidant capacity.

**Conclusion:**

miR-335-3p represents a potential independent prognostic marker in ESCC. The anti-tumor activity induced by miR-335-3p overexpression may be associated with its regulation of GFPT1.

## Introduction

The esophagus is a muscular tube that connects the pharynx to the stomach and primarily functions in food transport [1]. Esophageal cancer arises from the uncontrolled proliferation of esophageal cells, which gives rise to the formation of malignant tumors in the mucosal epithelium or glands [2]. Esophageal cancer constitutes 3.1% of newly diagnosed cancer cases globally and is mainly categorized into squamous cell carcinoma and adenocarcinoma [3, 4]. Among them, the former exhibits a strikingly high incidence in specific regions, such as East Asia and East Africa, reflecting distinct geographical and etiological patterns [5]. Given the esophagus’s intricate anatomical position, radical resection for esophageal cancer is both intricate and highly traumatic. Such surgery often leads to a variety of severe postoperative complications, and the rates of local recurrence and distant metastasis continue to pose significant clinical challenges [6].

In cancer drug research, E2F1 was believed to regulate the malignant phenotype of esophageal cancer cells by modulating the miR-29c-3p/COL11A1 signaling axis, thereby reducing the sensitivity of tumor cells to sorafenib [7]. Furthermore, P-hydroxylcinnamaldehyde has been reported to exert safe and effective antitumor effects against gastrointestinal tumors by modulating the PI3K/Akt and p53 apoptotic signaling pathways [8]. Similarly, the tumor suppressor TGFBR3 has been shown to inhibit the progression of lung cancer by regulating invasion, metastasis, and apoptosis, highlighting its potential as a therapeutic target [9]. Additionally, Vav1 protein has been found to be significantly associated with adverse clinical and pathological features and poorer survival rates in hepatocellular carcinoma [10]. Therefore, further exploration of the molecular mechanisms, therapeutic strategies, and prognostic biomarkers of esophageal cancer holds considerable potential for improving patient outcomes and alleviating the burden of this disease.

In the context of esophageal squamous cell carcinoma (ESCC), increasing evidence has highlighted the non-negligible role of oxidative stress in tumorigenesis and progression. Prolonged exposure of esophageal mucosa to various irritants may result in abnormal accumulation of reactive oxygen species (ROS), triggering lipid peroxidation [11]. This not only increases malondialdehyde (MDA) levels but also disrupts the dynamic balance of the intracellular antioxidant system. Recent findings have indicated that Gallic acid suppresses ESCC progression and enhances cisplatin chemosensitivity by promoting oxidative stress and inhibiting the IL-6/STAT3/Notch pathway [12]. Therefore, elucidating the molecular mechanisms regulating these specific oxidative stress indicators is important for understanding the pathological processes of ESCC.

MicroRNAs (miRNAs) represent a group of short, highly conserved RNA sequences in eukaryotes, with pivotal roles in modulating ontogeny and cell function [13, 14]. With the advancement of modern life sciences and medical research, an increasing number of reports indicate that the dysregulation of miRNAs is involved in the pathogenesis and progression of various tumors [15]. Taking esophageal cancer as an example, miR-497 has been believed to mediate treatment resistance and alleviate tumor progression by targeting QKI [16]. The miR-191-3p/RGS1 axis has been revealed to suppress the stemness of esophageal cancer cells by inhibiting the CXCR4/PI3K/AKT pathway [17]. miR-335-3p is located on chromosome 7q32.2 and has been identified as a tumor suppressor in multiple tumor types [18]. In triple-negative breast cancer, miR-335-3p was found to suppress cellular malignancy by targeting WNT2, and its downregulation is caused by the sponging effect of the lncRNA HAGLR [19]. Similarly, miR-335-3p was reported negatively expressed in acute myeloid leukemia, which inhibited cell proliferation and induced apoptosis by targeting and downregulating EIF3E in acute myeloid leukemia [20]. Furthermore, sequencing analysis also stated that miR-335-3p was downregulated in esophageal cancer cells [21]. Therefore, we hypothesized that miR-335-3p may mediate the pathological process of ESCC.

This study evaluated the prognostic significance of miR-335-3p by examining its quantitative expression and clinical correlations in 90 ESCC patients. The regulatory function of miR-335-3p, mediated via direct targeting of GFPT1, was clarified through functional assays in cancer cells. Our findings reveal a novel regulatory axis that may provide insights for future therapeutic strategies and prognostic evaluation in ESCC.

## Materials and methods

### Patients and clinical specimens

This retrospective study enrolled 90 patients with ESCC who received treatment at Jiujiang First People’s Hospital from September 2018 to March 2020. Eligible participants were required to satisfy the following criteria: the availability of comprehensive clinical and follow-up data; histopathological verification of ESCC by at least two board-certified physicians; newly diagnosed without any prior anti-cancer treatment; and the absence of other major comorbidities or a history of malignancy.

Surgical specimens, encompassing tumor tissues and paired adjacent normal tissues, were procured from patients undergoing esophagectomy. All tissue samples were promptly snap-frozen in liquid nitrogen and stored at − 80 °C until utilization.

The study protocol received approval from the Ethics Committee of Jiujiang First People’s Hospital, and written informed consent was acquired from each participant prior to the study.

### Follow-up for prognosis

All enrolled patients diagnosed with ESCC underwent a postoperative follow-up for a duration of five years. The follow-up for each participant was concluded either upon reaching the five-year endpoint or at the time of death, whichever event transpired first.

### RT-qPCR assay

RNA was extracted from specimens using TRIzol reagent (Invitrogen, USA). RNA that met the quality and concentration requirements underwent reverse transcription (TaqMan MicroRNA Reverse Transcription Kit) to generate cDNA. Subsequently, RT-qPCR amplification was carried out with the miScript SYBR^®^ Green PCR Kit (Qiagen, Germany) on an ABI 7900 real-time PCR system. Each sample was analyzed in triplicate and independently replicated at least three times. Data quantification was performed using the 2^−ΔΔCt^ method. U6 (for miR-335-3p) and GAPDH (for GFPT1) served as internal control.

### Cell culture

Human esophageal epithelial cells (SHEE10) and ESCC cells (KYSE-150, YES-2, TE-1, and TE-6) were sourced from ATCC. Specifically, KYSE-150 is a moderately differentiated ESCC cell line; YES-2 displays unique metastatic phenotypes; TE-1 and TE-6 are poorly differentiated lines with strong invasive capacity. These cell lines were cultured in DMEM supplemented with 10% fetal bovine serum (FBS; Gibco, USA) and incubated under standard conditions (37 °C, 5% CO₂). To maintain optimal growth and avoid overconfluence, the culture medium was replaced every 48 h, and cells were subcultured when they reached 80–90% confluence.

### Cell transfection

The overexpression constructs of miR-335-3p and GFPT1 (miR-335-3p mimic, oe-GFPT1) and matched negative controls were synthesized and provided by GenePharma (Shanghai, China). Cells were seeded into 6-well plates (2 × 10⁵/well) and allowed to reach 60–70% confluence before transfection. These constructs were transfected into YES-2 and TE-6 cells using lipofectamine 3000 reagent (Invitrogen, USA). Transfection efficiency was validated after 48 h.

### Cell biological activity assay

For cell proliferation assays, cells were seeded in 96-well plates (2 × 10^3^/well), with three replicate wells set up for each group and three biological replicates were performed. After 24 h, 10 µL of CCK-8 solution was added to each well for a total of 3 days. The optical density at 450 nm was measured after the addition of CCK-8 and incubation for 2 h.

For the invasion assay, the cell suspension (1 × 10⁵/well) was transferred to the upper of a Matrigel-coated Transwell chamber (BD, USA), with complete DMEM in the lower chamber. After 48 h, cells that invaded to the lower surface were stained with crystal violet. Cells were counted in at least five random microscope fields per chamber. For the migration assay, the Transwell chamber was used without Matrigel coating.

### Measurement of antioxidant indices

ROS and MDA levels were measured via Reactive oxygen species (ROS) Assay Kit (Beyotime, China) and Malondialdehyde (MDA) detection kit (Beyotime, China), respectively.

Cellular concentrations of glutathione (GSH) and oxidized glutathione (GSSG) were determined using GSH and GSSG detection kit (Beyotime, Shanghai, China), after which the GSH/GSSG ratio was calculated.

Samples were analyzed in triplicate, and all measurements were performed in three independent experiments.

### Luciferase reporter assay

Wild-type GFPT1 (WT-GFPT1) was amplified using the complementary sequences of miR-335-3p and GFPT1, whereas the mutant GFPT1 (MUT-GFPT1) was produced with mutated sequences. Both were cloned into the pmirGLO luciferase reporter plasmid. The above plasmids and mimic NC or miR-335-3p mimic were co-transfected into YES-2 and TE-6 cells by lipofectamine 3000 reagent, and the luciferase activity was evaluated at 48 h. The assay was repeated three times independently.

### Statistical analysis

Data processing and statistical analysis were conducted using GraphPad (9.0) and SPSS software (26.0). The normality of data distribution was assessed using the Shapiro-Wilk test, and homogeneity of variance was confirmed using Levene’s test prior to the application of parametric tests. Continuous variables that followed a normal distribution were presented as mean ± standard deviation (SD), and intergroup comparisons were made using t-tests or one-way ANOVA. Categorical data were expressed as counts (n) and analyzed with chi-square tests. To assess patient survival, Kaplan-Meier curves and Cox proportional hazards models were employed. Statistical significance was set at *P* < 0.05.

## Results

### Downregulation of miR-335-3p level in ESCC

**Fig. 1:**
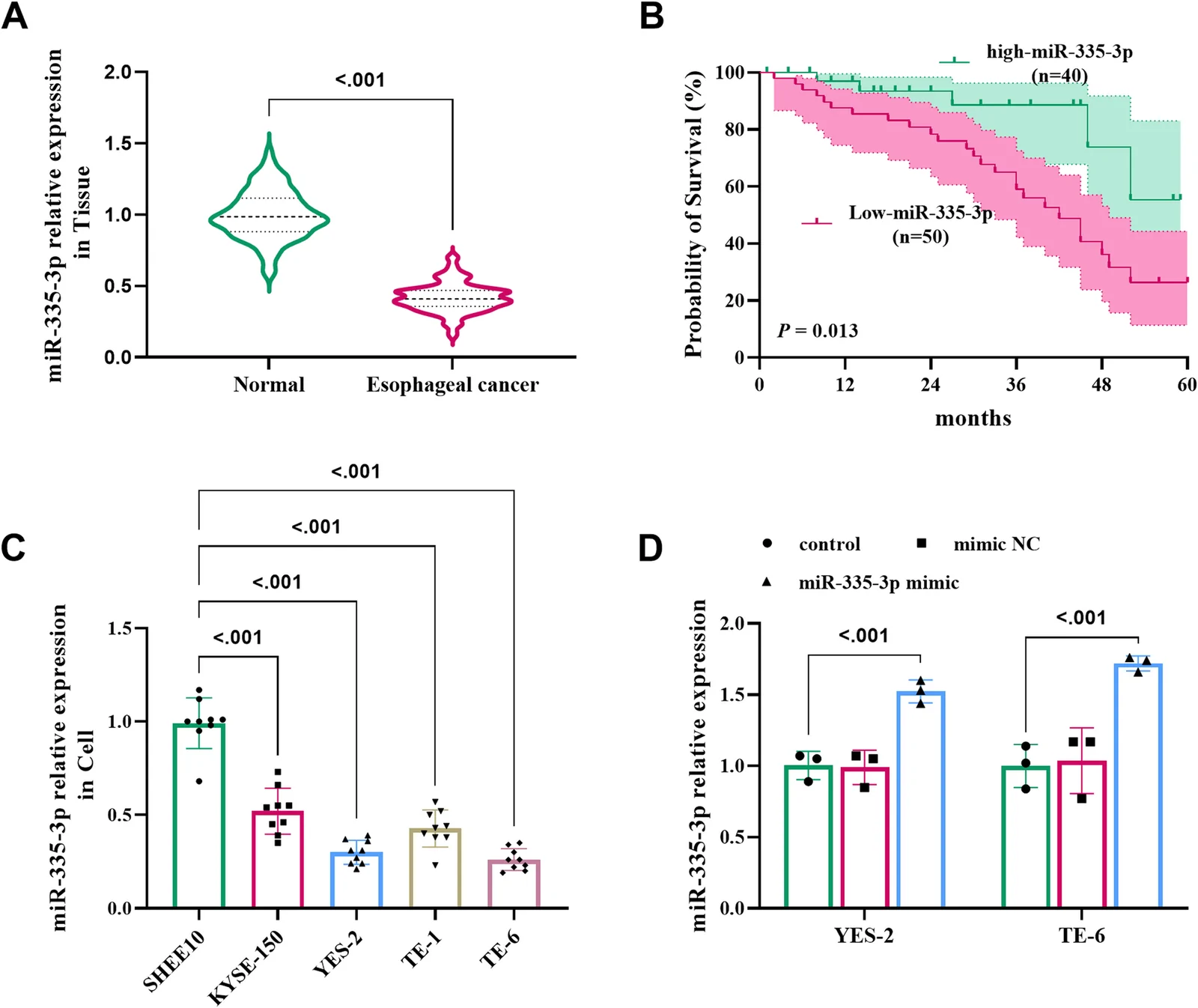
Silencing miR-335-3p indicates poor prognosis in ESCC. **A** miR-335-3p was downregulated in ESCC tissues. **B** The prognostic potential of dysregulated miR-335-3p in ESCC (*P* = 0.013). **C** miR-335-3p was lowly expressed in ESCC cells. **D** Transfection of miR-335-3p mimic enriched the miR-335-3p level in cells

A significant downregulation of miR-335-3p was observed in ESCC tissues relative to adjacent normal tissues (Fig. 1A). Using the mean expression value as a cutoff, patients were stratified into low and high miR-335-3p expression subgroups.

The examination of clinical indicators indicated that patients with low miR-335-3p expression exhibited more aggressive tumor characteristics, such as larger tumor size, lymph node metastasis, and advanced TNM stage (Table 1). Furthermore, ESCC patients with low-miR-335-3p exhibited poorer survival rates compared to those with high-miR-335-3p, as assessed by Kaplan-Meier curves (Fig. 1B). Meanwhile, multivariate Cox proportional hazards modeling identified miR-335-3p expression, lymph node metastasis, and TNM stage as crucial variables affecting patient survival outcomes (Table 2). The results indicate that suppression of miR-335-3p is associated with unfavorable prognosis in ESCC patients.

**Table 1:** Correlation between miR-335-3p expression and pathological characteristics of patients with ESCC

Characteristics	Cases (*n* = 90)	miR-335-3p expression		*P*
		Low (*n* = 50)	High (*n* = 40)	
Age				0.316
≤ 60 years	43	24	19	
> 60 years	47	26	21	
Gender				0.533
Male	53	28	25	
Female	37	22	15	
Tumor size				0.026
≤ 3 cm	40	17	23	
> 3 cm	50	33	17	
Lymph node metastasis				0.010
Negative	59	27	32	
Positive	31	23	8	
TNM stage				0.030
I-II	54	25	29	
III-IV	36	25	11	
Differentiation				0.266
Low	35	22	13	
Moderate or High	55	28	27	

**Table 2:** Multivariate Cox analysis for prognostic factors in patients with ESCC

Characteristics	HR	95% CI	*P*
miR-335-3p	0.209	0.065–0.668	0.008
Age	0.822	0.368–1.837	0.633
Gender	1.894	0.723–4.960	0.194
Tumor size	2.578	0.977–6.801	0.056
Lymph node metastasis	0.406	0.181–0.912	0.029
TNM stage	0.397	0.179–0.880	0.023
Differentiation	1.471	0.651–3.324	0.353

### miR-335-3p mimic exert an inhibitory effect on cellular activity

**Fig. 2:**
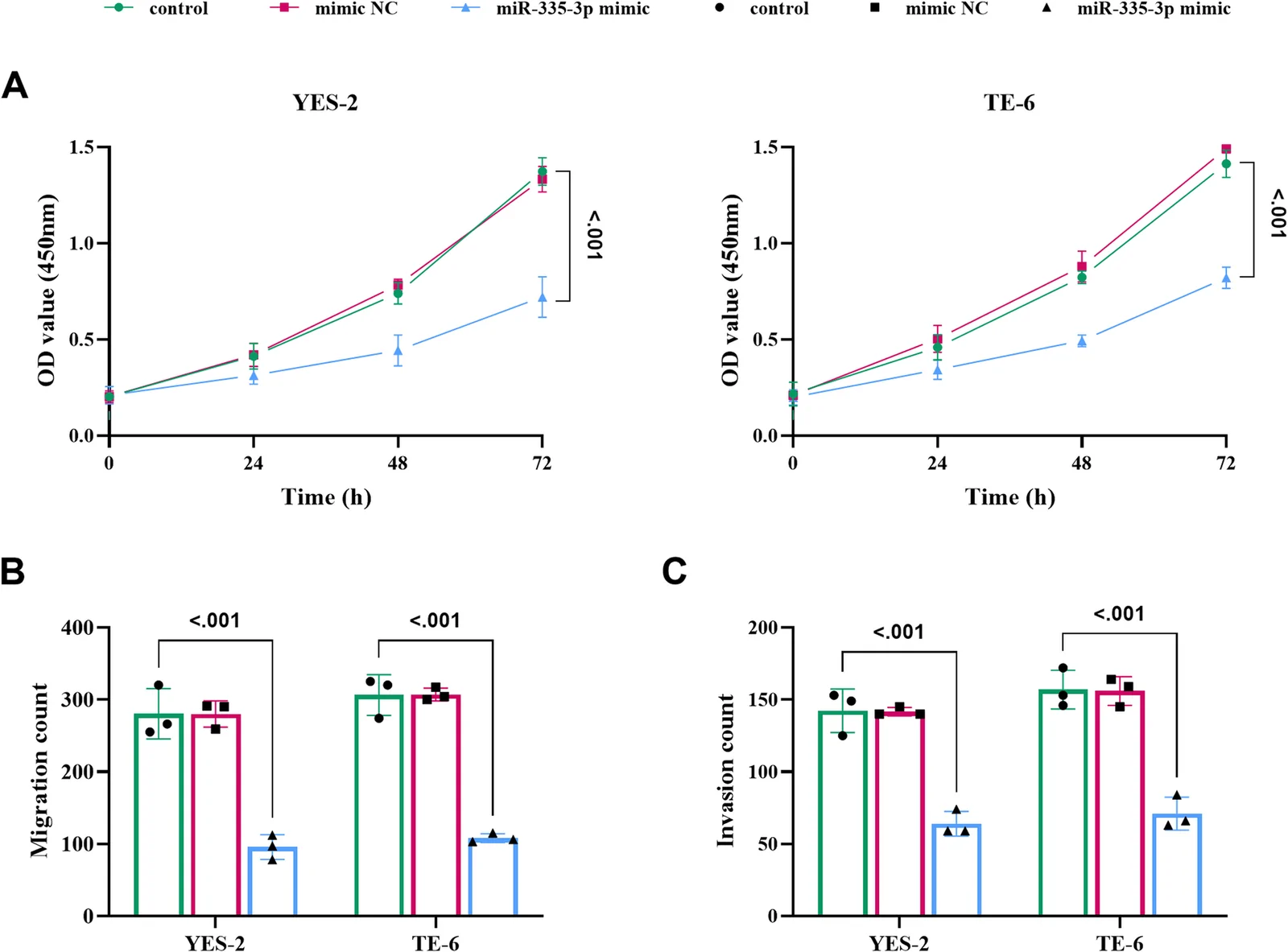
Upregulation of miR-335-3p mediated cell viability. **A** miR-335-3p mimic reduced the OD values in YES-2 and TE-6 cells. **B** miR-335-3p mimic affected the number of cell migration. **C** miR-335-3p mimic downregulated the cell invasion count

miR-335-3p level was reduced in ESCC cell lines KYSE-150, YES-2, TE-1, and TE-6 compared to normal SHEE10 cells, with the most pronounced reduction observed in YES-2 and TE-6 cells in Fig. 1C. Given this finding, the two cell lines were employed in subsequent functional assays. Transfection with miR-335-3p mimic markedly elevated miR-335-3p expression in YES-2 and TE-6 cells (Fig. 1D). When the level of miR-335-3p was increased, the proliferative capacity of cells was reduced (Fig. 2A), and the migration and invasion capabilities were also suppressed (Fig. 2B and C).

**Fig. 3:**
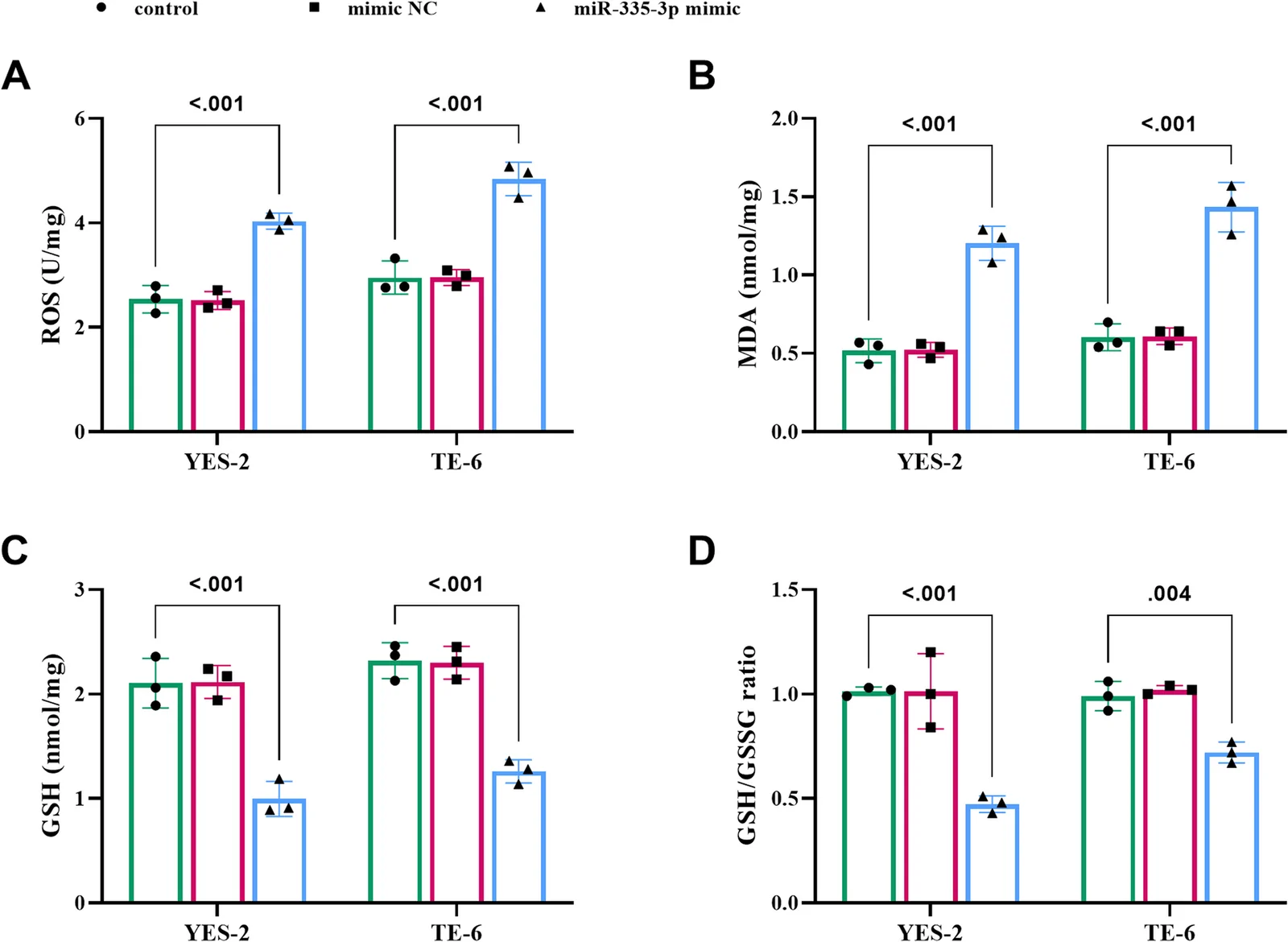
miR-335-3p mimic mediated oxidative stress response in cells. **A**-**B** Overexpression of miR-335-3p increased ROS and MDA content in the cells. **C-D** miR-335-3p mimic downregulated GSH levels and GSH/GSSG ratio in cells

Furthermore, miR-335-3p mimic enhanced the intracellular ROS level (Fig. 3A), and the MDA content was also increased (Fig. 3B), indicating that the upregulation of miR-335-3p enhanced oxidative stress levels in ESCC cells. Elevated miR-335-3p suppressed cellular antioxidant capacity by lowering GSH and GSH/GSSG ratio (Fig. 3C and D). These changes suggest that the overexpression of miR-335-3p is correlated with disrupted redox balance and the creation of an oxidative environment, which may be linked to the inhibition of malignant growth.

### GFPT1 was upregulated in ESCC

**Fig. 4:**
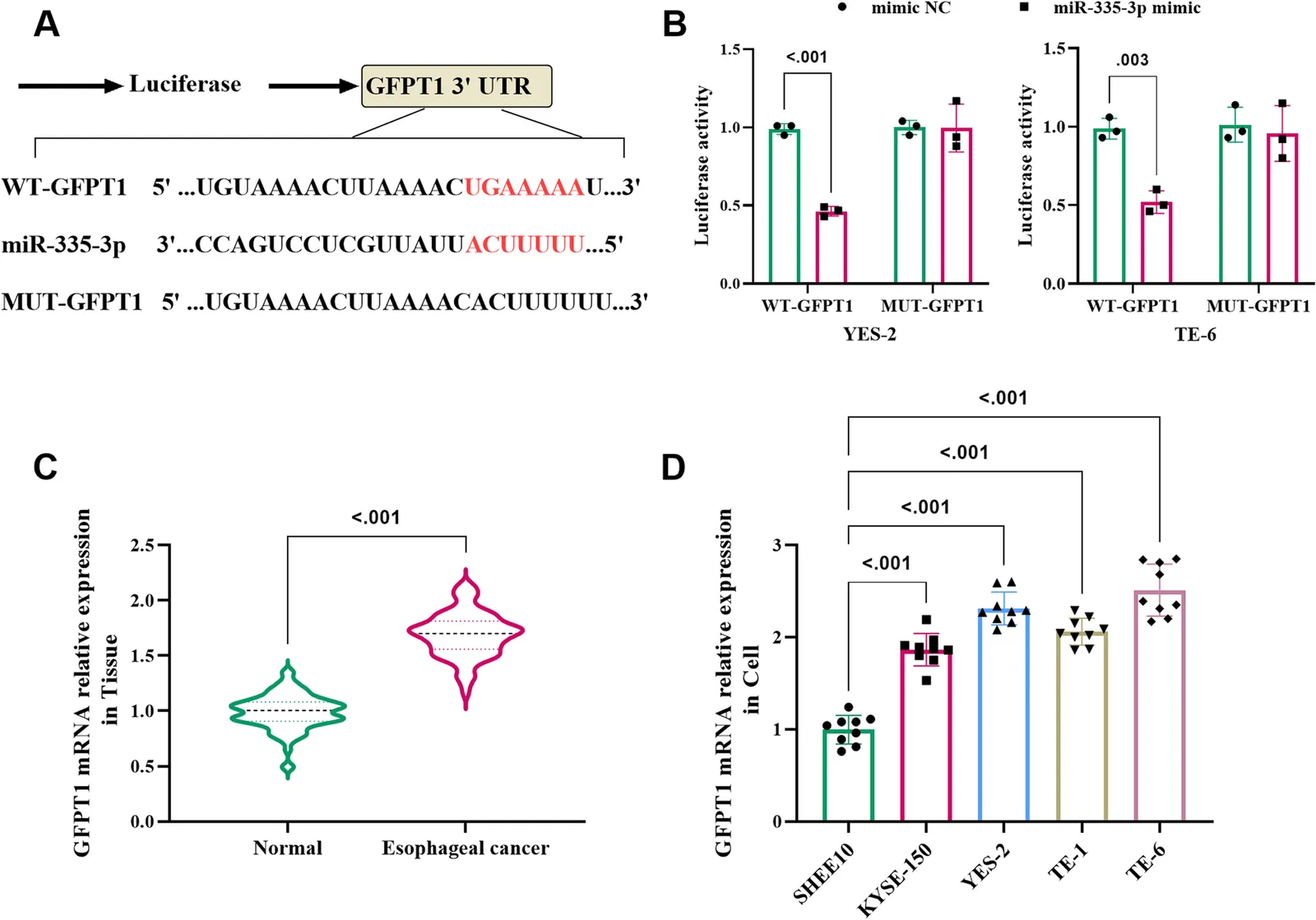
miR-335-3p targeted and regulated GFPT1. **A** TargetScan predicted that GFPT1 was the putative target of miR-335-3p. **B** The relationship between GFPT1 and miR-335-3p was verified by luciferase reporter assay. **C**-**D** GFPT1 mRNA was outstanding expression in ESCC tissues and cell samples

Bioinformatic analysis using TargetScan indicated that GFPT1 contains binding sites complementary to miR-335-3p, implying that it might act as a downstream target (Fig. 4A). Transfection of miR-335-3p mimic markedly suppressed luciferase activity in the WT-GFPT1 group within YES-2 cells, whereas no significant change was observed in the MUT-GFPT1 group. Comparable results were achieved in TE-6 cells (Fig. 4B). Quantitative assessment revealed that GFPT1 mRNA levels were obviously elevated in both tissue and cell samples (Fig. 4C and D).

### Upregulating GFPT1 partially counteracted the effects of the miR-335-3p mimic

**Fig. 5:**
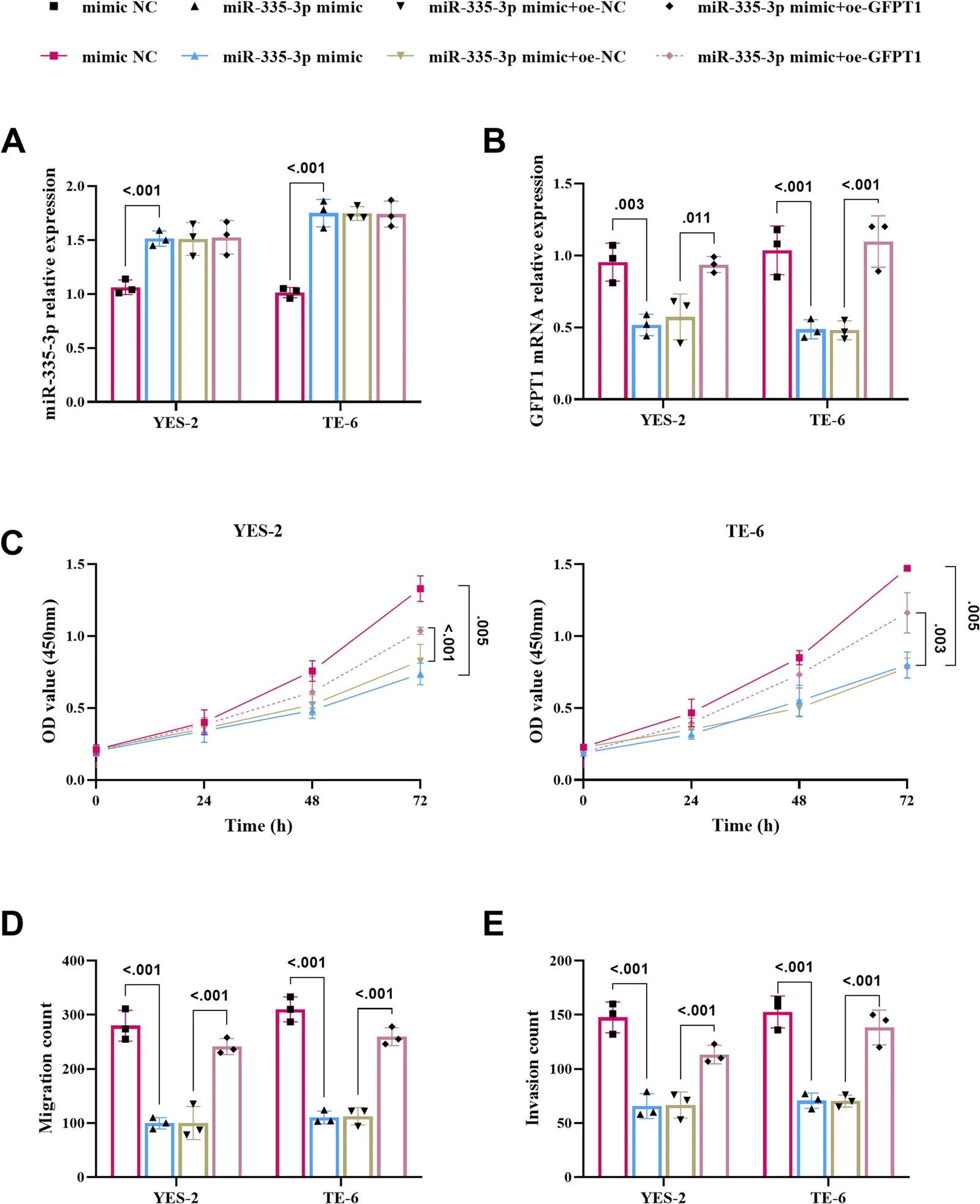
Regulation of cell biology functions by GFPT1 overexpression. **A**-**B** The effect of GFPT1 upregulation on the expression of miR-335-3p and GFPT1 in cells. **C**-**E** Upregulation of GFPT1 restored the malignant functions of the cells

Transfection of miR-335-3p mimic in YES-2 and TE-6 cells led to an upregulation of miR-335-3p expression and a downregulation of GFPT1 levels. Subsequent co-transfection with GFPT1 overexpression successfully rescued GFPT1 expression, while miR-335-3p levels remained unchanged (Fig. 5A and B). Additionally, co-transfection of miR-335-3p mimic and oe-GFPT1 partially recovered the cellular proliferation capacity (Fig. 5C). GFPT1 overexpression partially abolished the anti-migratory and anti-invasive effects induced by miR-335-3p mimic (Fig. 5D and E).

**Fig. 6:**
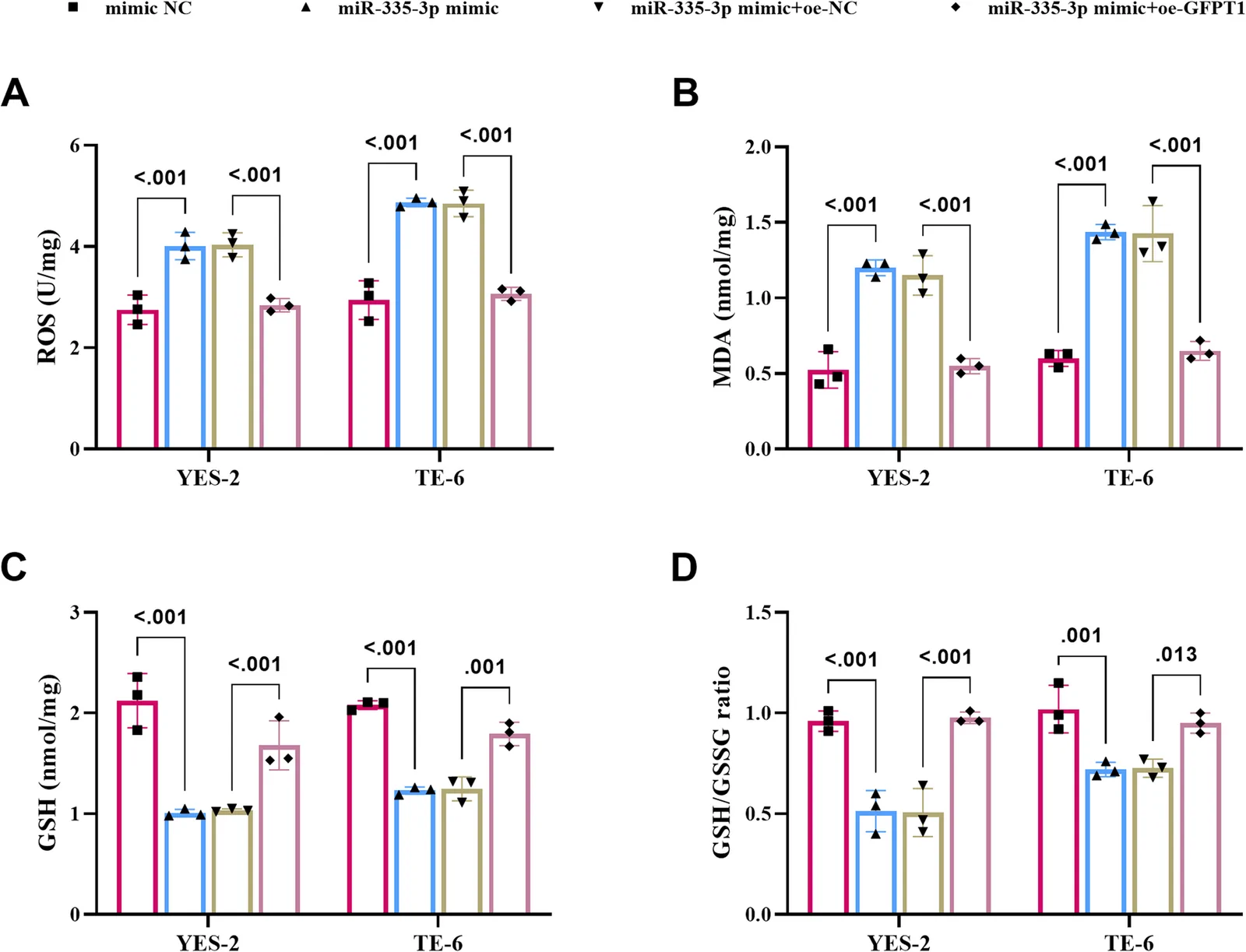
Regulation of oxidative stress by miR-335-3p/GFPT1 axis. **A**-**B** GFPT1 reversed the promotion effect of miR-335-3p on ROS and MDA levels in cells. **C**-**D** Transfection of oe-GFPT1 enhanced the antioxidant capacity of cells

In comparison with the miR-335-3p mimic group, levels of ROS and MDA were downregulated (Fig. 6A and B), whereas levels of GSH and the GSH/GSSG ratio were elevated in the miR-335-3p mimic + oe-GFPT1 group (Fig. 6C and D). GFPT1 overexpression reversed the abnormal oxidative stress induced by miR-335-3p and was closely related to the phenotypic rescue of ESCC malignant behavior, suggesting a potential association of the miR-335-3p/GFPT1 axis with the regulation of redox balance in ESCC.

## Discussion

The escalating incidence of ESCC substantially undermines patients’ quality of life. Given that the majority of ESCC cases are diagnosed at an advanced stage, tumor dissemination or metastasis persists as a frequent occurrence even after radical surgery, presenting the most formidable therapeutic challenge [22]. Consequently, identifying specific molecular targets for therapy and establishing reliable prognostic indicators are critical to improving patient outcomes.

miRNAs possess a pleiotropic nature, enabling them to regulate multiple genes and signaling pathways by targeting numerous sites on their mRNA transcripts, which underlies their diverse biological functions [23]. For instance, miRNAs possess intricate regulatory networks, thereby producing different pathophysiological changes in many diseases [24]. miR-27a-3p, miR-485-5p, miR-203, and miR-26a-5p have all been reported to exhibit abnormal expression in esophageal cancer [25–28]. miR-335-3p has been documented to be diminished in type II diabetes, diabetic retinopathy, and T-cell acute lymphoblastic leukemia [29–31]. Similarly, miR-335-3p was also detected with low expression in ESCC tissues in this study. Notably, miR-335-3p may act as an oncogenic factor in certain contexts, such as its high expression in rhabdomyosarcoma, which is associated with patient survival [32]. This highlights the specificity of miRNA function. Downregulated miR-335-3p was strongly associated with larger tumor size, lymph node metastasis, and advanced TNM stage, leading to unfavorable prognosis and diminished survival rates. Among the various clinicopathological parameters, lymph node metastasis and TNM staging have been recognized as major prognostic indicators [33]. Interestingly, we observed a study highlighting the critical role of molecular dysregulation in the ESCC microenvironment and the importance of identifying novel regulatory factors for prognostic assessment [34]. Mechanistically, we found that miR-335-3p was attenuated in ESCC cells, and miR-335-3p mimic effectively controlled cell growth and migration. Interestingly, Zhang et al. ‘s latest report suggested that miR-100 also inhibits the behavior of tumor cells by mediating signaling pathways including PI3K/AKT and Wnt/β-catenin [35], which can be used as our future research direction. This reinforces our observation that miR-335-3p is a key tumor suppressor, positioning it as a promising independent prognostic marker for ESCC.

GFPT1 was verified to be a direct downstream target of miR-335-3p and was overexpressed in ESCC. GFPT1 was also demonstrated to be overexpressed in pancreatic ductal adenocarcinoma and cervical cancer [36, 37], consistent with the trend of the present study. GFPT1 is a rate-limiting enzyme in the hexosamine biosynthesis pathway (HBP), which redirects glucose metabolism toward the production of UDP-glucuronic acid, reprogramming oncogenic signaling through protein glycosylation, enhancing stress tolerance, and meeting the biosynthetic demands of cancer cells [38]. Therefore, GFPT1 is considered a core molecule linking cellular metabolism to malignant cancer behavior [39]. Zhang et al. pointed out that GFPT1 is a direct target of miR-183-3p in esophageal cancer, and its level enrichment partially rescued the function of lncRNA ELFN1-AS1 [40]. Additionally, GFPT1 expression was markedly enhanced in breast cancer, promoting immune evasion by upregulating PD-L1 expression [41]. In the present study, the increase of GFPT1 counteracted the inhibitory effect of miR-335-3p mimic on the proliferation function of YES-2 and TE-6 cells. Similarly, the migratory and invasive properties of cells were also reversed by co-transfection of GFPT1 and miR-335-3p.

Notably, these alterations were manifested not only functionally but also through the improvement of the cellular oxidative stress status. It was observed that upregulation of miR-335-3p increased ROS and MDA levels, and reduced GSH content and the GSH/GSSG ratio, whereas GFPT1 restored the oxidative stress parameters. This result reveals the crucial role of the miR-335-3p/GFPT1 axis in regulating the redox balance in tumor cells. The decrease of GSH content and the GSH/GSSG ratio in cancer cells may indicate that ferroptosis is activated, which will significantly downregulate the activity of tumor cells [42]. Consistent with recent metabolic insights in ESCC, Zheng et al. reported that metabolic reprogramming mediated by MCT4 drives a pro-tumorigenic acidic microenvironment [43]. Based on the observed correlation, this finding suggests that miR-335-3p targeting GFPT1 may disrupt the metabolic adaptation mechanisms that maintain tumor cell survival and redox homeostasis. From a therapeutic perspective, a previous study revealed that CXCL6 upregulates PD-L1 by activating STAT3 pathway to accelerate ESCC progression and metastasis [44]. Given this, miR-335-3p, which inhibits ESCC malignant behavior, also holds promise as a potential component of combination therapy targeting metabolic pathways and immune checkpoints such as PD-L1.

Despite our confirmation of the molecular mechanism and prognostic value of miR-335-3p in ESCC, the current study remains constrained by several factors. One major limitation is that mechanistic studies rely on in vitro cell models, which fail to fully recapitulate the complexity of the human tumor microenvironment or the systemic effects observed in vivo. Subsequent studies could validate the regulatory role of miR-335-3p using animal models to bridge the gap between in vitro experiments and clinical relationships. Secondly, our clinical analysis was based on a single cohort of patients without external validation, which may restrict the broader applicability and generalizability of our findings to the wider ESCC population. Addressing this issue requires large-scale, multicenter longitudinal cohort studies to confirm the expression and prognostic function of miR-335-3p in different populations. Furthermore, while GFPT1 was identified as a key target, the intricate upstream regulatory networks involving miR-335-3p remain to be fully elucidated. Finally, implementation in esophageal cancer management remains in its early stages, and widespread adoption requires phased clinical trials targeting molecular pathways to determine the feasibility of using miR-335-3p to treat ESCC.

In conclusion, miR-335-3p level is obviously reduced in ESCC and serves as an independent prognostic indicator of poor survival. Restoration of miR-335-3p inhibits cancer cell progression, a process that may be mediated by the downregulation of GFPT1. These findings provide a theoretical basis for miR-335-3p as a promising candidate for a prognostic biomarker and a targeted therapeutic strategy.

## Data Availability

The datasets used and/or analysed during the current study are available from the corresponding author on reasonable request.
